# Carbon Aerogels: Synthesis, Modification, and Multifunctional Applications

**DOI:** 10.3390/gels11070548

**Published:** 2025-07-15

**Authors:** Liying Li, Guiyu Jin, Jian Shen, Mengyan Guo, Jiacheng Song, Yiming Li, Jian Xiong

**Affiliations:** 1 School of Ecology and Environment, Xizang University, Lhasa 850000, China; liliyinglly1101@163.com (L.L.); greglee@foxmail.com (Y.L.); 2 College of Environment and Resources, Xiangtan University, Xiangtan 411105, China; js_xtuhzy@xtu.edu.cn; 3 School of Environmental Science and Engineering, Tianjin University, Tianjin 300350, China; mengyanguopublic@tju.edu.cn

**Keywords:** carbon aerogels, electrochemistry, water treatment and adsorption

## Abstract

Amidst global imperatives for sustainable energy and environmental remediation, carbon aerogels (CAs) present a transformative alternative to conventional carbon materials (e.g., activated carbon, carbon fibers), overcoming limitations of disordered pore structures, unmodifiable surface chemistry, and functional inflexibility. This review systematically examines CA-based electrochemical systems as its primary focus, analyzing fundamental charge-storage mechanisms and establishing structure–property–application relationships critical to energy storage performance. We critically assess synthesis methodologies, emphasizing how stage-specific parameters govern structural/functional traits, and detail multifunctional modification strategies (e.g., heteroatom doping, composite engineering) that enhance electrochemical behavior through pore architecture optimization, surface chemistry tuning, and charge-transfer kinetics acceleration. Electrochemical applications are extensively explored, including the following: 1. Energy storage: supercapacitors (dual EDLC/pseudocapacitive mechanisms) and battery hybrids. 2. Electrocatalysis: HER, OER, ORR, and CO_2_ reduction reaction (CO_2_RR). 3. Electrochemical processing: capacitive deionization (CDI) and electrosorption. Beyond this core scope, we briefly acknowledge CA versatility in ancillary domains: environmental remediation (heavy metal removal, oil/water separation), flame retardancy, microwave absorption, and CO_2_ capture.

## 1. Introduction

The escalating depletion of fossil fuels, coupled with the alarming accumulation of greenhouse gases and the accelerating degradation of global ecosystems, underscores the critical need for sustainable energy solutions. To mitigate anthropogenic climate impacts and achieve carbon neutrality, the development of high-performance electrochemical energy storage systems is paramount. Renewable energy integration demands technologies capable of balancing high power density, rapid charge–discharge kinetics, and extended cycle stability. Conventional energy storage devices often face limitations in power density and safety, while traditional capacitors lack sufficient energy density. Supercapacitors, bridging this gap through dual mechanisms of electric double-layer capacitance (EDLC) and pseudo-capacitance, have emerged as pivotal candidates for grid-scale energy storage, electric vehicles, and portable electronics [1,2,3,4,5]. However, optimizing their performance while aligning with environmental sustainability—through eco-friendly materials and scalable synthesis—remains a pressing challenge. 

Carbon aerogels, a class of three-dimensional (3D) porous nanomaterials, epitomize versatility in various applications due to their unique hierarchical architecture and tunable physicochemical properties. Defined by IUPAC as non-fluidic networks of interconnected colloidal particles with gas-dominated porosity, carbon aerogels exhibit exceptional attributes, including ultra-high specific surface area, low density, continuous conductive frameworks, and mechanical robustness [6]. Among all types of aerogels, carbon aerogel is the only conductive aerogel and is an ideal electrode material for preparing supercapacitors and catalyst carriers [7,8,9,10]. Derived from pyrolysis of organic precursors (e.g., resorcinol–formaldehyde gels), their 3D crosslinked nanostructure facilitates rapid ion diffusion and electron transport, making them ideal for EDLC-dominated supercapacitors. In addition, their chemical stability, heat resistance, and compatibility with functionalization (e.g., heteroatom doping, metal oxide recombination [11,12,13,14]) make it possible to enhance the synergistic pseudo-capacitance. The basic principles of capacitors involved in energy storage applications and the relationship between different application requirements and CAs performance characteristics [1,11,15,16] will be introduced in detail in Section 2 of this manuscript. However, the raw materials of most carbon aerogels depend on the petroleum industry, are expensive, cause great harm to organisms and the environment, and the raw materials are mostly non-flexible brittle solids, and the microstructure is difficult to control [17]. Unlike petroleum-derived precursors, emerging sustainable feedstocks (e.g., biomass [18], polymers) are now being explored to address environmental concerns associated with conventional synthesis, further aligning carbon aerogels with green chemistry principles.

Focusing on research advances over the past decade, this review systematically investigates fundamental capacitive mechanisms in energy storage applications, elucidating the correlation between diverse application requirements and the performance characteristics of carbon aerogels (CAs). Subsequently, it critically examines contemporary synthesis methodologies of CAs, with emphasis on how process parameters at distinct preparation stages influence their structural and functional properties. The remainder of this review comprehensively analyzes recent multifunctional modification strategies achieved through various doping approaches and their derivative applications. As a lightweight porous functional material, carbon aerogel has been widely explored in emerging fields such as capacitors, catalysis, capacitive deionization, oil/water separation, removal of heavy metal ions, flame retardancy, heat insulation, microwave absorption, and CO_2_ capture [19,20,21,22,23,24] due to its unique three-dimensional network structure, large specific surface area, low density, excellent heat resistance, high conductivity, and mechanical stability [25,26,27,28,29,30,31,32,33,34,35,36,37].

## 2. Basics of Capacitive Fundamentals of Various Applications and Correlations Between Each Application and Carbon Aerogel Properties

Supercapacitors are a new type of green energy storage devices, possessing advantages such as high energy density, high specific power, long service life, fast charge–discharge speed, and high cycling efficiency [17,38,39]. Supercapacitors can be classified into two categories according to the energy storage principle. One is the electric double-layer capacitor (EDLC), which stores capacitance by utilizing the double layer formed at the interface between the surface of the electrode material with a high specific surface area and the solution due to electrostatic attraction. Carbon-based materials, such as activated carbon, carbon fiber, carbon nanotube, and carbon aerogel, are commonly used as electrode materials for this type of energy storage method [2,3,4,5,40,41,42]. The other category is pseudo-capacitance, which utilizes electrochemical deposition or the redox Faradaic process. Metal oxides and conductive polymer materials are commonly used as electrode materials for this energy storage mode [11,12,13,14,43,44]. The former realizes energy storage through a physical process relying on electrostatic attraction, while the latter achieves energy storage through chemical adsorption and desorption by means of redox chemical reactions. To meet the various requirements of electronic devices for supercapacitors (SCs), different types of electrode materials are used in new SCs. Among numerous electrode systems, carbon-based materials, mainly including multiple types such as graphene, carbon nanotube, carbon fiber, carbide derivatives, MXene, carbon aerogel (CA), and activated carbon are regarded as the best electrode active materials [45,46,47,48,49,50]. Among them, carbon aerogel (CA) has become an ideal electrode material for supercapacitors due to its flexibility, high specific surface area, chemical inertness, controllable porosity, and excellent electrical conductivity.

### 2.1. Double-Layer Capacitors

**Figure 1:**
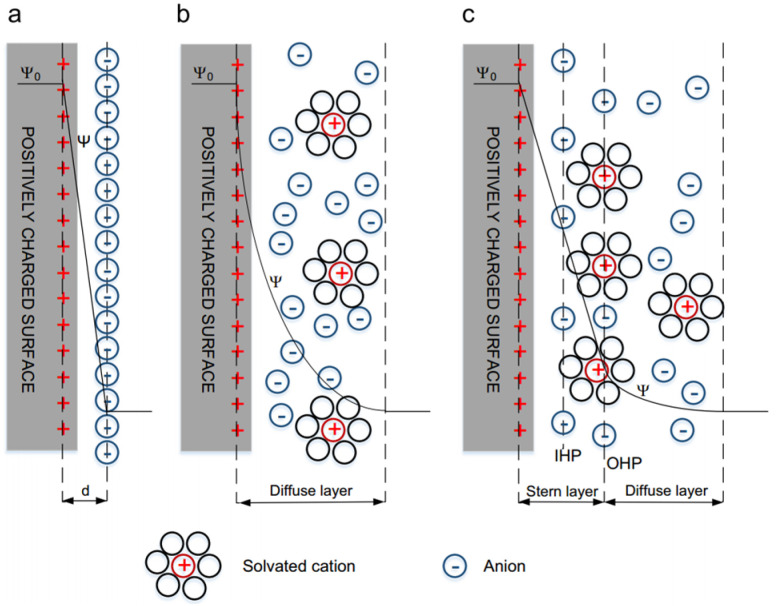
EDL models: (**a**) Helmholtz model, (**b**) Gouy–Chapman model, and (**c**) Stern model. Reprinted with permission from [1].

Electric double-layer capacitors, store energy by utilizing the double layer formed at the interface between the electrode material and the electrolyte. When a conductor comes into contact with an electrolyte, stable double layers of charges with opposite signs will be generated at the interface, thus achieving the purpose of energy storage. Such double layers of charges are called electric double layers. The principle of the electric double layer was first discovered by the famous German physicist Helmholtz. The structure of the electric double layer is shown in Figure 1.

The electric double layer is composed of two opposite-charge layers with a distance of atomic size. These two charge layers are just like the two plates of a traditional capacitor. When an external voltage is applied to the two plates of a supercapacitor, just like an ordinary capacitor, the positive plate of the capacitor stores positive charges and the negative plate stores negative charges. Under the action of the electric field generated by the charges on the two plates, opposite-charge layers will be formed at the interface between the electrolyte and the electrode. During charging, the electrode surface becomes charged, and the ions with opposite charges in the electrolyte move towards the two poles, respectively, and are closely arranged on the electrode surface. During discharging, electrons move from the negative electrode of the active material to the positive electrode, and positive and negative ions return from the electrode surface to the electrolyte. As the supercapacitor discharges, the charges on the positive and negative plates are discharged through the external circuit, and the charges at the interface between the electrode and the solution decrease accordingly. This indicates that the capacitance of the electric double layer is directly proportional to the specific surface area of the electrode. Therefore, the purpose of increasing the energy stored in the capacitor can be achieved by increasing the specific surface area of the electrode material. Moreover, this charging and discharging process is always a physical process and does not involve chemical reactions, so it has stable performance and a long cycle life. Sandhiya M et al. [2] made the first attempt to synthesize N-doped porous activated carbon (PW) from poultry manure for the manufacture of flexible supercapacitors. In the experiment, using PW material as the raw material, N-doped porous activated carbon was successfully synthesized by the chemical activation method. After chemical activation with KOH, the specific capacitance increased by 7.2 times. After the activation of the PW material, the energy density of the supercapacitor increased exponentially, significantly increasing from 16 Wh/kg to 23 Wh/kg. In addition, the durability of the supercapacitor after activation increased from 83% to 99%. The activated PW-derived carbon has a relatively high specific surface area, thus improving the specific capacitance and energy density. Due to the high degree of graphitization, the cycle life is extended. The all-solid-state flexible supercapacitor prepared using nitrogen-doped activated carbon exhibits a huge energy density of 21.5 Wh/kg. Qian et al. [2] embedded structural carbon fiber fabrics into the continuous network of carbon aerogel (CAG) to form a coherent yet porous whole. The addition of CAG significantly increased the surface area of the carbon fiber fabric, so the electrochemical performance was improved by more than 100 times. The CAG-normalized specific electrode capacitance measured in an aqueous solution by cyclic voltammetry was approximately 62 F/g. Using an ionic liquid electrolyte, after introducing CAG into the carbon fiber fabric, the estimated energy density increased from 0.003 to 1 Wh/kg. Two CAG-carbon fabrics were sandwiched around an insulating separator to form a functional structural double-layer electrochemical capacitor composite. The modification of CAG not only increased the electrochemical surface area but also strengthened the polymer around the fibrils, thereby significantly improving the performance of the material.

### 2.2. Pseudo-Capacitors

In the redox process occurring in conductive polymers, the generation of current is achieved through the infiltration and release of ions in the electrolyte within the polymer skeleton. This process not only takes place on the polymer surface but also runs through the entire polymer system and has a high degree of reversibility. However, due to the swelling and contraction phenomena that occur in conductive polymers during the charging and discharging process, their mechanical properties will deteriorate, resulting in a significant attenuation of their electrochemical performance [3].

**Figure 2:**
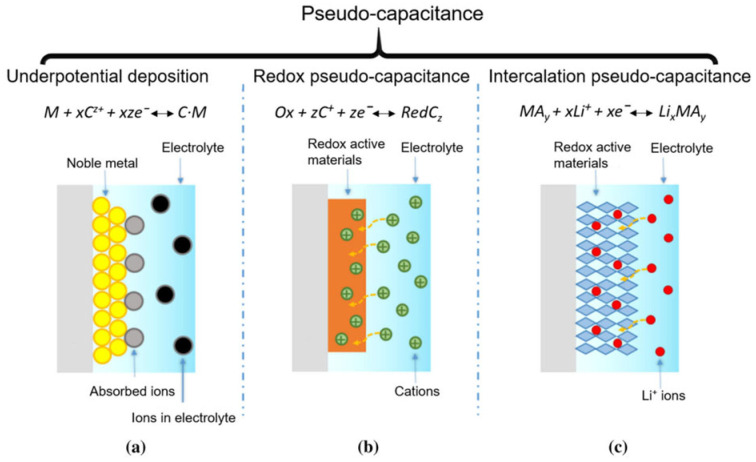
Schematics of charge storage in pseudo-capacitors: (**a**) underpotential deposition, (**b**) redox reactions, (**c**) ion insertion/extraction. Reprinted with permission from [4].

The Faradaic pseudo-capacitor is also known as the pseudo-capacitor. The principle of its energy storage is shown in Figure 2.

The electric double-layer capacitor mainly stores charges through electrostatic adsorption on the electrode surface, and no redox reactions occur in the electrode material. In contrast, for the pseudo-capacitor, in the two-dimensional or quasi-two-dimensional space on the electrode surface or in the bulk phase, electroactive substances undergo underpotential deposition and highly reversible chemisorption/desorption or redox reactions, thereby generating a capacitance that depends on the charging potential of the electrode [5,6,7]. The Faradaic pseudo-capacitance generated on the electrode surface has a completely different charge storage mechanism from that of the electric double-layer capacitor. The electrode materials of pseudo-capacitors mainly fall into two categories. One category is transition metal oxides, such as RuO_2_ [8], MnO_2_ [9], Fe_3_O_4_ [10], etc. Their energy storage mechanism is that in the electrode surface or in the bulk phase, electroactive substances undergo underpotential deposition and highly reversible chemisorption/desorption or redox reactions. Moreover, these reactions can penetrate deep into the interior of the electrode. Therefore, energy is stored in a three-dimensional space, and they possess a high energy density. The other category is conductive polymers, such as polyaniline [11] polypyrrole [12], polythiophene [13], etc., mainly utilizing their ability to dope and dedope charges. Their energy storage mechanism is that, through rapid and reversible n-type and p-type element doping and dedoping redox reactions occurring in the polymers on the electrodes, the polymers can store charges at a high density, thus generating a high Faradaic pseudo-capacitance. Song et al. [14] designed a multifunctional honeycomb-like nitrogen-doped carbon/bimetallic sulfide and oxide composite aerogel through simple mechanical mixing, freeze-drying, and heat treatment methods. The prepared N-CoFe_2_O_4_-CoxSy/FexSy@C composite material has excellent electromagnetic wave absorption performance and electrochemical performance. The specific capacitance of this material is 2883 F/g at 1 A/g, and its cycling stability after 5000 cycles is 89.5%, which is attributed to abundant active sites and a well-defined heterogeneous structure.

## 3. Synthesis Protocols of Carbon Aerogels

As a new type of aerogel, compared with the traditional silica aerogel, carbon aerogel has more remarkable properties, such as higher porosity, a smaller diameter of network colloidal particles, larger specific surface area, and lower high-temperature thermal conductivity, etc., which also endows carbon aerogel with broader application prospects. Therefore, the preparation of carbon aerogel and the methods to improve its performance have attracted the attention of many researchers.

### 3.1. Synthesis and Characterization of Pure Carbon Aerogel Materials

**Figure 3:**
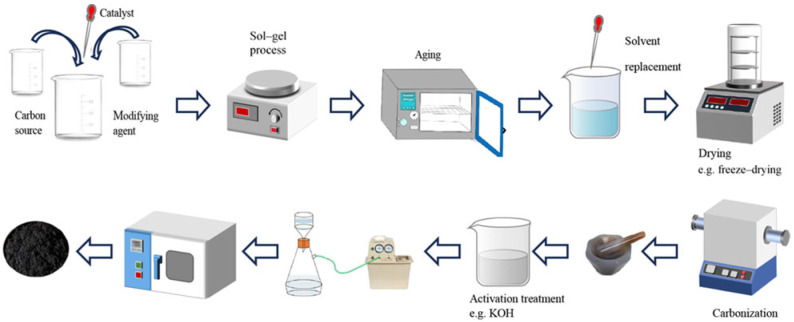
The preparation process of carbon aerogel.

Carbon aerogel was first prepared by Pekala et al. [15] from Lawrence Livermore National Laboratory in the United States in 1989. They used resorcinol (R) and formaldehyde (F) as raw materials, synthesized by the sol–gel method [16], and further carbonized to obtain RF carbon aerogel (CAs) with a three-dimensional network structure. Since the successful development of carbon aerogel, its preparation methods have been continuously updated. The selection of raw materials and catalysts, change in reactant ratios, and improvements in the preparation process have all significantly improved the performance of carbon aerogel. Although the preparation methods of carbon aerogel are changing with each passing day, its main preparation process has not changed much, mainly including steps such as the sol–gel process, solvent replacement and drying, carbonization, and activation treatment. The preparation process is roughly shown in Figure 3.

1. Sol–gel process: The sol–gel process is the basic reaction for the formation of the three-dimensional skeleton precursor of the aerogel. Resorcinol (R) and formaldehyde (F) are stirred evenly under the action of an alkali catalyst (usually sodium carbonate) to fully carry out addition and condensation reactions, thereby forming a polymer with a network structure. Then, the polymer is placed in a closed container for aging treatment, and finally, the RF organic wet gel is obtained.

During the reaction process, factors affecting the structure and performance of carbon aerogel, such as reactant concentration [17], reactant ratio [18], catalyst concentration [19,20], and reaction temperature [21], should be strictly controlled. The electrical conductivity of carbon aerogel depends on the bulk density of carbon aerogel, and an important factor affecting the bulk density is the change in mesopore volume. The larger the mesopore volume, the smaller the bulk density, and the higher the electrical conductivity. The reactant concentration determines the mesopore volume of carbon aerogel. The reactant ratio (the molar ratio of resorcinol to formaldehyde, R/F) also affects the mesopore volume of carbon aerogel. When R/F ≤ 0.34, the mesopore structure of carbon aerogel may be damaged, and when R/F ≥ 0.7, it may cause insufficient gelation and an inability to dry. The specific surface area and mesopore volume of carbon aerogel will first increase and then decrease with the increase in the catalyst concentration (the molar ratio of reactants to catalyst, R/C), and its pore structure will also gradually become uniform. Therefore, the catalyst concentration should be adjusted to the optimum value. Excessively high reaction temperatures and overly short reaction times may cause insufficient gelation, so the reaction is generally carried out at room temperature (20 °C). Adjusting the pH value of the solution between 5.4 and 7.6 is conducive to the progress of the condensation reaction.

2. Solvent replacement and drying: After the aging process of the wet gel is completed, solvent replacement is carried out. A low-surface-tension solvent is used to replace the liquid in the pores to reduce the capillary stress, thereby preparing an aerogel with an intact structure. This is to avoid the structural collapse caused by shrinkage during the subsequent drying process. The solvents generally used for replacement are non-aqueous solvents (such as methanol, ethanol, isopropanol, and acetone, etc.), and the replacement is repeated several times until the inorganic solvent is completely replaced. After solvent replacement, the wet gel can be dried by various drying methods to obtain a solid aerogel.

- **Drying treatment of the organic gel:** Under atmospheric conditions, the conventional evaporation of the solvent may cause a drastic change in surface tension when the vapor–liquid interface is formed. The huge difference in the surface tension between the coexisting gas phase and liquid phase generates significant mechanical stress, leading to the collapse of the pore structure. To ensure the porous structure of the aerogel, the drying process needs to be carried out without affecting the microstructure of the wet gel. Depending on the gel system, methods such as subcritical, supercritical, freeze-drying, vacuum drying, and atmospheric drying can be selected [22,23,24,25,26,27].
- **Atmospheric drying:** Atmospheric drying represents the most common and scalable method for aerogel production, enabling large-scale manufacturing as evidenced by the successful synthesis of hydrophobic silica aerogels in the following example. This technique facilitated the creation of aerogels with functional properties critical for applications: Khedkar et al. [28] achieved super hydrophobicity (0~154°), low density (0.12 g/cm^3^), and a high surface area (792 m^2^/g), while in their subsequent research, Khedkar et al. [29] further demonstrated the tunability of physicochemical properties (density: 0.10–0.18 g/cm^3^; surface area: 538–802 m^2^/g) through pH variation during sol–gel processing.
- However, inherent limitations arise from nanoscale pore structures, where capillary forces during solvent evaporation induce significant microstructural stress. This results in partial network collapse and a reduced specific surface area, as observed in the variable textural properties in Khedkar’s 2020 study [29] (surface area reduction to 538 m^2^/g at suboptimal pH) and the thermal stability threshold (478 °C) in his 2019 study [28]. Despite these constraints, optimized conditions (e.g., pH = 5 in Khedkar’s 2020 study [29]) can yield aerogels with balanced performance in transparency, thermal stability (536 °C), and surface area (802 m^2^/g), validating the method’s practicality for industrial adoption despite structural compromises.
- **Subcritical drying:** Subcritical drying is carried out when the mechanical strength of the gel is sufficient to withstand the capillary pressure (sometimes under atmospheric pressure), which can avoid significant structural changes. Although air drying is faster, simpler, and cheaper than the supercritical or subcritical carbon dioxide extraction and drying process, it may lead to the shrinkage of the pore structure of the xerogel.
- **Freeze-drying:** The framework structure of the cryogel after freeze-drying remains relatively intact. Freeze-drying is to remove the solvent by sublimation after freezing the solvent to avoid the formation of the gas–liquid interface, but it may still lead to the shrinkage of the gel. Moreover, it is important to perform solvent exchange (such as replacing with tert-butanol) before freeze-drying the wet gel to prevent the gel structure from being damaged and large pores from being generated due to the expansion of the aqueous solution.
- **Supercritical drying:** If the specific surface area of the aerogel is to be further increased, supercritical drying is currently the best choice [30]. Supercritical drying can maintain the network structure of the aerogel well. The process of supercritical drying involves filling the air with liquid CO_2_, exchanging the solvent for a low-surface-tension solvent such as CO_2_, and making it enter the supercritical state to minimize the mechanical stress on the pore walls. The aerogel prepared by this method has outstanding characteristics but is sensitive to synthesis conditions, and the high-pressure time is long. Although supercritical drying with an organic solvent such as acetone can shorten the time, the shrinkage degree and density of the aerogel are larger and the color is darker, and the shrinkage rate is sensitive to the depressurization rate.

Comparing the above drying methods, it is found that the supercritical drying method has the best drying effect. It not only avoids the capillary action between the pores of the carbon aerogel during the drying process, reduces the deformation of the carbon aerogel, and maintains the original framework structure, but the obtained product also has a higher porosity and specific surface area compared with the other two gels.

3. Carbonization: The carbonization of organic aerogel [31] requires calcining the dried aerogel at a high temperature under an inert atmosphere or vacuum conditions (Table 1) to remove the oxygen-containing and hydrogen-containing functional groups in it and convert it into the corresponding carbon aerogel. During the carbonization process, conditions such as carbonization temperature, heating rate, and carbonization time need to be strictly controlled. Kim et al. [32] increased the final carbonization temperature and decreased the heating rate in an N_2_ environment, effectively controlling the density of the carbon aerogel (0.6 g/cm^3^) to remain unchanged and maximizing its electrical conductivity (≈50 S/cm).

4. Activation treatment: The activation treatment of carbon aerogel can optimize its pore structure and surface morphology, improve its performance, and thus meet specific application requirements. The number of micro- and mesopores of the carbon aerogel material can be increased through physical or chemical activation treatment methods. In fact, any activation method applied to activated carbon can in principle be applied to the activation treatment of carbon aerogel. Commonly used activation methods include physical activation (generally carbon dioxide activation [35]) and chemical activation (generally KOH activation [36]). Commonly used treatment methods are shown in Table 2, including the steam activation method, carbon dioxide activation method, phosphoric acid activation method, potassium hydroxide activation method, zinc chloride activation method, etc. [37]. Carbon dioxide activation can maintain the mesopore structure of carbon aerogel microspheres, which is beneficial for the ingress and egress of ions or electrons inside, while KOH activation is conducive to the formation of micropores in carbon aerogel and can increase its electrochemically active surface area. These activation methods can effectively improve the electrochemical performance of carbon aerogel, and the specific capacity of the activated carbon aerogel increases several times.

5. Purification and impurity removal: Carbon aerogels derived from different carbon sources, such as biomass (e.g., watermelon rind, lignin) or synthetic precursors, often contain impurities, including (1) inorganic residues (catalysts (KOH) and activators (ZnCl_2_)); (2) organic tar (byproducts of incomplete carbonization); and (3) ash content (silicates/metals from biomass). Common impurity removal methods include the solvent exchange step mentioned earlier (e.g., ethanol), which not only reduces surface tension but also utilizes organic solvents to remove organic residues. Another method involves acid washing the prepared carbon materials with hydrochloric acid solution, which helps remove metal ions and silica, as well as reduce the ash content, which is critical for electrochemical performance.
6. Characterization: Instrumental techniques are commonly employed to characterize carbon aerogels, evaluating their various properties and structural features. The advantages and limitations of representative characterization methods are summarized in Table 3 below.

### 3.2. Modification of Carbon Aerogel Materials

**Figure 4:**
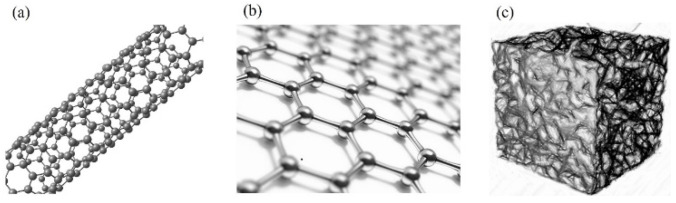
Physical models of common carbon-based materials: (**a**) one-dimensional carbon nanotubes; (**b**) two-dimensional graphene; (**c**) three-dimensional carbon aerogel.

Compared with carbon materials such as one-dimensional carbon nanotubes and two-dimensional graphene, carbon aerogel has a continuous three-dimensional porous network structure [45,46], as shown in Figure 4. However, the single carbon aerogel electrode material has the disadvantages of low energy density, small specific capacitance, and small specific capacity, which limit its large-scale application in the electrochemical field [47]. Since the performance of a single carbon aerogel is average and cannot yet meet the performance requirements for commercialization, researchers have attempted to improve its performance through modification. Currently, the modification methods mainly fall into two categories: one is to introduce heteroatoms (such as N, S, P elements) to adjust the particle size and improve the utilization rate of porosity and specific surface area; the other is to combine carbon aerogel with graphene materials, conductive polymer materials, and transition metal compound materials and utilize the excellent conductive properties of the materials to improve the relevant performance of the overall composite material and further enhance the electrochemical performance of the carbon aerogel material.

#### 3.2.1. Heteroatom Doping (N, P, S, etc.)

During the modification process of heteroatom-doped carbon aerogels, the introduction of heteroatoms significantly regulates the pore structure and chemical environment of adsorption sites through multiple mechanisms. The pore characteristics of materials are jointly influenced by the atomic size of dopants, doping procedures, and precursor properties. For instance, porosity collapse may occur in carbon aerogel during heteroatom doping, which may be caused by the following conditions:

- Atomic Size and Spatial Occupancy: Heteroatoms (e.g., P, S) exhibit larger atomic radii than carbon (P: 195 pm, S: 180 pm vs. C: 170 pm), leading to steric hindrance during doping. For instance, phosphorus doping initially expands micropores or generates mesopores by occupying additional spatial volume [48], but excessive P loading exerts compressive stress on the carbon skeleton, causing pore wall collapse. Sulfur doping, particularly via high-temperature pyrolysis, induces up to 80% volumetric expansion due to sulfur sublimation, which disrupts the porous network [49]. Structural defects (e.g., vacancies, edge sites) formed during high-temperature carbonization facilitate micro/mesopore creation initially, but excessive doping (e.g., sulfur-induced expansion) can conversely collapse or block pores [49].
- Thermal Stress during High-Temperature Carbonization: Heteroatom doping often involves high-temperature processes (e.g., >600 °C for N-doping with urea), where heteroatoms induce structural defects that initially promote pore formation [49]. However, excessive doping enhances thermal instability, causing uneven shrinkage of the carbon matrix. For example, Song et al. [14] found that deviating from a 1:2 Co^2+^/Fe^3+^ ratio in N-CoFe_2_O_4_ composites led to uneven thermal expansion, resulting in pore collapse.
- Redox Reactions and Functional Group Interactions: Heteroatoms alter the carbon matrix’s electronic distribution, promoting oxygen-containing functional groups (e.g., carboxyls, phenols) [50]. During activation (e.g., KOH treatment), these groups undergo redox reactions, generating gaseous byproducts (CO, CO_2_) that exert internal pressure on pores. For instance, KOH activation of N-doped carbon aerogels at 800 °C produces H_2_O and CO_2_, which may collapse micropores if gas evolution exceeds pore diffusion capacity [40].
- Synergistic Effects with Activation Processes: Chemical activation (e.g., KOH) combined with heteroatom doping exacerbates pore collapse. KOH etching preferentially removes carbon atoms adjacent to heteroatom sites, weakening pore walls. Li et al. [51] showed that PPy-grafted N-doped carbon aerogels under KOH activation had 15% lower pore volume than undoped counterparts due to combined etching and heteroatom-induced stress. Moderate nitrogen doping increases specific surface area via micropore generation, but excessive doping or synergistic activation can reverse this effect [49].

Regarding the chemical environment, heteroatoms directly alter the electronic distribution of the carbon matrix and the nature of surface functional groups, thereby influencing adsorption site activity and interfacial behavior. For example, the main role of nitrogen doping in carbon electrode materials is to improve the nature of the surface functional groups of the carbon material, thereby enhancing the wettability between the carbon material and the electrolyte, further improving the utilization rate of pores and specific surface area, and contributing part of the pseudo-capacitance to improve its charge storage capacity [50]. In addition, nitrogen atoms, as electron donors, provide more electrons for the delocalized carbon atoms and increase the conductivity of the carbon material [52]. Besides increasing wettability and conductivity, the most important role of nitrogen doping in carbon-based electrocatalysts is to form catalytic active sites. For example, in the application of electrocatalytic oxygen reduction reaction, pyridine-N atoms are considered to be effective active sites [53].

There are mainly two methods for heteroatom-doped carbon aerogel (taking nitrogen atom doping as an example [54]). One is in-situ doping, such as preparing nitrogen-doped carbon aerogel using chitosan as the raw material. The other is post-treatment doping, such as preparing nitrogen-doped carbon aerogel by high-temperature carbonization of cellulose and urea or doping carbon aerogel by soaking it in a urea solution.

**Figure 5:**
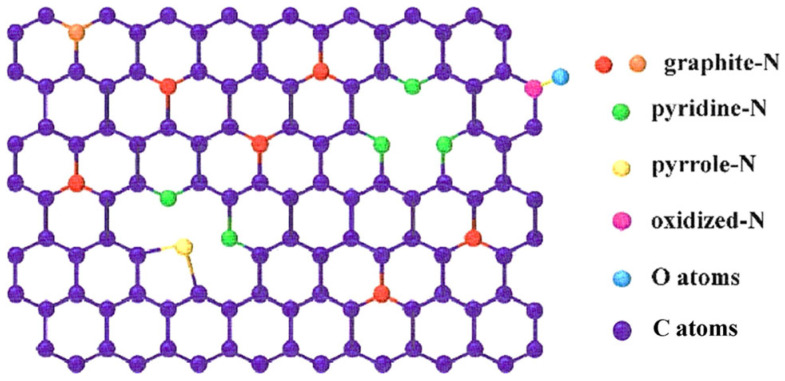
Doping forms of N atoms in the carbon material.

Nitrogen atoms combine with the sp^2^ hybridized carbon lattice network to form four forms of nitrogen atom bonding states (as shown in Figure 5): pyridine-N, pyrrole-N, graphite-N, and oxidized-N. The changes in the physical and chemical properties of the carbon material caused by nitrogen doping enhance both its energy storage and electrocatalytic performance (Table 4 below).

Chen et al. [66] developed a milk protein–surfactant (SDS)–graphene micelle system and prepared a graphene-based aerogel by hydrothermal and freeze-drying methods. The decrease in micelle size in this system can adjust the new surface properties of the aerogel, thereby increasing the specific surface area. Subsequently, the aerogel was further graphitized and activated to prepare N-doped porous nanocarbon at 600 °C. The optimized nitrogen-doped nanocarbon material (MGPC-5) has an enhanced specific surface area and degree of graphitization, and exhibits excellent capacitive performance and stability in both the KOH three-electrode system (518.8 F/g at a current density of 0.1 A/g) and the symmetric electrode system (120.8 F/g at a current density of 0.1 A/g, with a capacitance retention rate of 95% after 5000 charge–discharge cycles at 3 A/g).

Carbon aerogel with excellent conductivity is highly favored in the fields of electrochemical energy storage and energy conversion catalytic materials, and the high conductivity of carbon materials is related to the high degree of graphitization. In the energy storage field, a low resistivity can reduce the internal resistance of the device and improve the Coulombic efficiency, and fast charge transfer can improve the rate performance of the energy storage device. There is an inter-constraint relationship among the carbonization temperature, the degree of graphitization, the specific surface area, and the functional groups. Protein carbonization at high temperatures can generate conjugated sp^2^ carbon atoms to increase the degree of graphitization; the lower the carbonization temperature, the lower the degree of graphitization and the worse the conductivity; ultra-high temperature carbonization will cause the collapse and disappearance of pores and functional groups due to corrosion, reducing the specific surface area [67], all of which will reduce the electrochemical performance of carbon aerogel. Therefore, a balance among the degree of graphitization, pore structure, specific surface area, and nitrogen doping amount is required to maximize aerogel performance. To achieve this balance, transition metal (Fe, Co, Ni, etc.)–assisted carbonization can effectively improve the degree of graphitization of carbon aerogel. Lee et al. [68] prepared carbon aerogel via the condensation polymerization of resorcinol and formaldehyde at room temperature using sodium carbonate as a catalyst. Nickel-doped carbon aerogel nanoparticles (21.0%, 35.0%, 60%, and 82%) were prepared in an ethanol solvent by the precipitation method, and their performance on supercapacitor electrodes was studied. The Faradaic redox reaction of nickel oxide nanoparticles enhanced the capacitance of the carbon aerogel nanoparticles. Using cyclic voltammetry, under the conditions of a scanning rate of 10 mV/s and constant current charge–discharge in 6 M KOH electrolyte at 1 A/g, the 35 wt% Ni-doped carbon aerogel (Ni/CA-35) exhibited the highest capacitance (110 F/g) and excellent charge–discharge performance, and it also exhibited fairly stable cycling performance, indicating long-term electrochemical stability. Song et al. [69] synthesized nitrogen-doped hierarchical porous carbon (NHPC) extracted from waste protein sericin and used it in an electrochemical capacitor for the first time. The prepared NHPC has a specific surface area of 2723 m^2^ g^−1^ and a pore volume of 1.42 cm^3^ g^−1^, and it has a high specific capacitance of 287 F/g at a current density of 0.5 A/g, which is comparable to or even higher than that of many previously reported porous carbon materials. Using this electrode, about 93% of the initial capacitance (221 F/g at 5 A/g) remained unchanged after 10,000 cycles, exhibiting good cycling stability. The specific capacitance of sericin-derived NHPC is also better than that of sericin-derived porous carbon.

#### 3.2.2. Conductive Polymer Grafting

Conductive polymers (e.g., polyaniline, polypyrrole, polyindole, polythiophene) exhibit excellent pseudo-capacitive behavior through redox reactions on their conjugated chains, combined with a large specific surface area [70]. Fast and reversible n-type/p-type adsorption/desorption redox reactions enable these polymers to generate high Faradaic pseudo-capacitance for electrical energy storage [71]. When compounded with carbon aerogels, their properties complement each other to form ideal electrode materials.

However, conductive polymers in supercapacitor electrodes cause mechanical stability issues due to significant volume changes (up to 30%) during charge–discharge cycles from ion intercalation/deintercalation [3]:

- Framework Degradation: Repeated swelling/contraction weakens the polymer–carbon aerogel interface, leading to delamination. Zhang et al. [72] found that PPy/CA composites with >50 wt% PPy showed 20% capacitance decay after 10,000 cycles due to structural fatigue.
- Pore Blocking: Polymer aggregation in mesopores (10–50 nm) reduces ion accessibility. Khammar et al. [73] observed that low PANI loadings (<20 wt%) blocked micropores in CX aerogels, decreasing the specific surface area from 1200 to 850 m^2^/g.

Zhang et al. [72] produced a homogeneous core-sheath polypyrrole/carbon aerogel (PPy/CA) composite via the in-situ oxidative polymerization of pyrrole on a structurally robust and compressible carbon aerogel made from bamboo cellulose nanofibers. Attributed to the synergy between the high power density of CA and the high energy density of PPy pseudo-capacitance, this composite capacitor can provide a specific capacitance of 268.5 F/g at a current density of 0.5 A/g and has excellent power density and energy density, as well as ultra-high cycling stability (the capacitance retention rate reaches 88% after 10,000 cycles at 10 A/g). Li et al. [51] prepared different proportions of polypyrrole (PPy)/nitric acid-activated carbon aerogel (HCA) composites by chemical oxidative polymerization. Their study shows that through nitric acid activation and compounding with polypyrrole, the porous morphology of the carbon aerogel was not damaged. Both the nitric acid-activated carbon aerogel and the polypyrrole/nitric acid-activated carbon aerogel still maintained the original three-dimensional nanoporous structure of the carbon aerogel. When the ratio of polypyrrole to nitric acid-activated carbon aerogel was 1:1, the composite exhibits the optimal electrochemical performance: the specific capacitance is as high as 336 F/g, which is more than three times that of the pure carbon aerogel (103 F/g). In addition, it also exhibited excellent conductivity and cycling stability, with 91% of the initial capacitance remaining after 2000 cycles, possessing excellent properties as a supercapacitor electrode material. Qiu et al. [74] prepared a series of heteroatom-doped carbon materials with hierarchical porous and ultra-high specific surface area (up to 3673 m^2^/g) by using a simple chemical foaming strategy during the pyrolysis process of poly(bisphenoxy)phosphazene (PBPP). The ideal pore structure combined with N, P, and O co–doping enabled the material to have a specific capacitance of 225.0 F/g in a symmetric cell at 0.5 A/g. The excellent pore structure made the specific capacitance of polyaniline grown in situ on its surface as high as 413.1 F/g at a current density of 1 A/g.

Among all conductive polymers, polyaniline can be used as a good electrode material due to its easy synthesis, high environmental stability, reversible redox, and low cost [75]. Shang et al. [76] utilized the unique three-dimensional network of cellulose-derived carbon aerogel (CA), coated polyaniline (PANI) on its surface as a scaffold framework to anchor ZIF-8, and successfully synthesized a new type of three-dimensional cross-coupled hierarchical porous carbon composite material. This material had small micropores (0.89 nm), wide mesopores/macropores (10–80 nm), a large specific surface area (347 m^2^/g), and rich N self-doping (6.27 wt%). Due to the synergistic advantages of the porous structure of CA, the N doping and wettability of the PANI layer, and the micropores and double-layer capacitance of ZIF-8, the prepared ZPCA electrode had a high specific capacitance, excellent rate performance, and good cycling stability, thus exhibiting excellent electrochemical performance. Khammar et al. [73] explored the influence of different polyaniline loadings on the porosity and related electrochemical properties of the composite by the in-situ polymerization of aniline using a hierarchical porous carbon xerogel (CX) as the basic skeleton through the sol–gel method and chemical oxidative polymerization. It was found that a low loading of PANI blocked almost all the CX micropore structures, reducing its specific surface area. Increasing the PANI loading would develop a mesopore structure in the CX layer. This composite (PANI/CX) exhibited a high specific capacitance (451 F/g) and relatively excellent cycling stability (the capacitance retention rate reached 87.6% after 1500 repeated cycles). The doping of conductive polymers with carbon aerogel not only improved the structure and wettability of the carbon aerogel but also helped to generate pseudo-capacitance.

#### 3.2.3. Carbon Aerogel/Transition Metal Compound Composites

Transition metal compound electrode materials, such as Co(OH)_2_ [77], Co_3_O_4_ [78], ZnO [79], MnO_2_ [80], NiCo_2_O_4_ (nickel cobaltate) [81], NixCo_3_-xSy (nickel cobalt sulfide) [82], Fe_3_O_4_ [62], SnO_2_ [83], NiS [84], etc., are popular due to their excellent electrochemical properties such as high specific capacitance and energy density. However, due to problems such as low electrical conductivity, insufficient mass transfer, and attenuation of the critical capacity at high current densities, the wide application of transition metal-based electrode materials is limited to a certain extent [85].

The enhancement of electrochemical performance in transition metal compound-doped carbon aerogels is primarily attributed to the synergistic effects between the unique electronic structures of transition metal compounds and the carbon matrix. Transition metals (e.g., Fe, Co, Ni, Mn) and their oxides, sulfides, or nitrides typically exhibit variable valence states, abundant d-orbital electrons, and catalytically active sites. These properties, when integrated with carbon aerogels to form heterojunction structures, significantly optimize interfacial electron transfer kinetics [81,86,87,88]. For instance, at heterojunction interfaces, differences in Fermi levels between transition metal compounds and the carbon matrix induce interfacial charge redistribution, generating localized built-in electric fields that accelerate directional charge migration and reduce interfacial resistance [5,89]. This effect is particularly critical in energy storage or catalytic processes. In supercapacitors, for example, heterojunction interfaces facilitate rapid electron transfer from the carbon matrix to transition metal active sites, while the high conductivity of certain metal compounds further enhances the efficiency of the overall electrode’s conductive network [90].

Additionally, unsaturated coordination atoms or defect sites (e.g., oxygen vacancies, sulfur vacancies) on the surface of transition metal compounds act as highly active sites, strengthening the adsorption and activation of reactive intermediates such as OH^−^, H^+^, or polysulfides [91,92]. Furthermore, the introduction of transition metal nanoparticles may suppress the structural collapse of the carbon matrix during cycling via a “spatial confinement effect” and optimize mechanical stability by regulating local graphitization [10,93]. However, an excessive aggregation of transition metals may shield active sites or block pores, necessitating precise control of doping concentration and dispersion to achieve optimal performance [85,94]. Therefore, more and more researchers are keen on modifying transition metal-based electrode materials (the modification of porous carbon materials is shown in Table 5).

One modification strategy is to combine transition metal compounds with a three-dimensional conductive framework and utilize the synergy between the two for modification. Dong et al. [82] used waste zucchini peel as a carbon source and prepared carbon aerogel (CA) loaded with NixCo_3_-xSy nanoparticles using hydrothermal and calcination methods, and tested the electrochemical properties of NixCo_3_-xSy /CA under five different Ni: Co ratios. The research showed that the electrochemical performance of the NiCo_2_S_4_/CA composite was the best. α-CA was prepared with K_2_FeO_4_ as a pore-forming agent. α-CA has a three-dimensional (3D) hierarchical porous structure and a large specific surface area, thereby improving the cycling stability and electrical conductivity. The asymmetric supercapacitor has a maximum energy density of 33.8 Wh/kg at a power density of 800 W/kg and a long cycling life of 87.4% after 10,000 cycles at 10 A/g. Yang et al. [102] found that metal oxide nanoparticles with a smaller particle size can expose more active sites for reaction with the electrolyte. Therefore, they used N, P–co–doped carbon materials with rich nanopores and an ultra-high surface area as a new material to limit the growth of Co_3_O_4_ nanoparticles. Such composite modification prepared a Co_3_O_4_/carbon composite material with ultrasmall (<3 nm) Co_3_O_4_ firmly anchored. The composite material exhibited excellent capacitive performance (1310 F/g at 0.5 A/g) and good cycling stability (92% capacitance retention rate after 5000 cycles). The excellent electrochemical performance of the carbon aerogel/transition metal-based composite material indicates that this modification and composite method has great potential in the practical application of supercapacitors.

### 3.3. Comparison of Different Preparation Methods

The preparation method of pure carbon aerogel mentioned above and several other different types of doping methods each have their own advantages. Table 6 compares the different preparation methods.

Discrepancies in the performance of carbon aerogels (CAs) resulting from different preparation methods stem from multiple experimental variables. Electrolyte composition critically influences electrochemical behavior, as evidenced by N-doped CAs exhibiting a specific capacitance of 225 F/g [55] in 1 M H_2_SO_4_ versus 281 F/g in 6 M KOH [56]. This divergence is attributed to differential ion mobility (H^+^ < K^+^) and pH-dependent surface charge modulation. Furthermore, characterization methodologies introduce significant variations: cyclic voltammetry (CV) systematically overestimates specific capacitance compared to galvanostatic charge–discharge (GCD) techniques. Representative CV measurements report 387.6 F/g at 2 mV/s [64], exceeding GCD-derived values due to scan-rate-dependent polarization effects. Critically, precursor selection fundamentally governs material properties. Lignin-derived CAs achieve only 189 F/g [57], whereas cellulose-derived counterparts attain 328 F/g [65], reflecting intrinsic structural limitations of lignin’s cross-linked aromatic networks versus the ion-accessible linear polymeric chains in cellulose.

### 3.4. Reproducibility in Synthesis of Carbon Aerogels

#### 3.4.1. Key Parameters and Reproducibility Challenges in Synthesis Processes

The reproducibility of carbon aerogel synthesis is influenced by multi-stage parameters, with core steps including sol–gel reaction, drying, carbonization, and activation. In the sol–gel process, reactant concentration (e.g., the resorcinol–formaldehyde system), molar ratio (R/F), catalyst concentration (R/C), and reaction temperature must be strictly controlled. For instance, when R/F ≤ 0.34, the mesoporous structure of carbon aerogels may be damaged; when R/F ≥ 0.7, insufficient gelation leads to drying failure [18]. The catalyst concentration (e.g., Na_2_CO_3_) must be optimized to the optimal value; otherwise, the specific surface area and mesopore volume first increase and then decrease [19,20]. If the reaction temperature exceeds room temperature (20 °C) or the reaction time is too short, gelation insufficiency may occur [21].

In the drying stage, supercritical drying maintains the network structure but is sensitive to pressure and temperature, and the decompression rate affects the shrinkage ratio [30]. Freeze-drying requires solvent exchange (e.g., tert-butanol) to avoid structural damage caused by ice crystal expansion, and improper operation leads to pore collapse [22,23,24,25,26,27]. During carbonization, temperature, heating rate, and atmosphere (e.g., N_2_ protection) significantly impact product density and electrical conductivity. For example, Kim et al. maintained the density of carbon aerogels (0.6 g/cm^3^) and maximized electrical conductivity (≈50 S/cm) in N_2_ by increasing the carbonization temperature and decreasing the heating rate [32], but minor fluctuations in these conditions may cause performance variations.

#### 3.4.2. Impacts of Raw Material Sources and Pretreatment

Traditional petroleum-based raw materials (e.g., resorcinol) have stable properties but suffer from high cost and environmental hazards. Biomass-based raw materials (e.g., cellulose, lignin) exhibit reduced synthesis reproducibility due to differences in sources (e.g., plant species, growth conditions), components (cellulose/hemicellulose/lignin ratios), and impurity contents. For instance, carbon aerogels prepared from winter melon and lettuce show fluctuations in density and hydrophobicity due to differences in biomass pretreatment (e.g., washing, crushing) [104,105]. Additionally, unwashed metal ions (e.g., Na^+^) in biomass may block pores (e.g., sodium salt residues in LTCA), affecting the pore structure of subsequent carbonized products [106].

#### 3.4.3. Reproducibility Bottlenecks in Modification Processes

In heteroatom doping and composite modification, doping amount and dispersibility are critical. For example, in nitrogen doping, variations in urea soaking concentration or high-temperature carbonization time lead to fluctuations in N content, thereby affecting electrochemical performance. The specific capacitance of the N-CoFe_2_O_4_-CoxSy/FexSy@C composite aerogel prepared by Song et al. [14]. (2883 F/g) depends on the metal salt ratio and heat treatment temperature. Deviation of the Co^^2^+^/Fe^^3^+^ ratio from 1:2 results in non-uniform active site distribution and performance dispersion [14]. During conductive polymer (e.g., PPy) grafting, the degree of polymerization and coating uniformity are influenced by reaction time and oxidant concentration. In the PPy/HCA composite aerogel prepared by Li et al., the PPy loading must be strictly controlled at 1:1 to avoid pore blockage [51].

### 3.5. Stability of Carbon Aerogel Materials

Carbon aerogels exhibit remarkable mechanical stability, attributed to their three-dimensional porous network structure, which allows them to withstand external forces without significant deformation. For instance, supercritical drying methods help maintain the network structure, enhancing mechanical robustness [30]. Additionally, composite modifications with materials like conductive polymers or transition metal compounds further strengthen their mechanical properties [72,93].

In terms of electrochemical stability, carbon aerogels demonstrate excellent performance in supercapacitor applications, with high capacitance retention rates over numerous cycles. For example, N-doped carbon aerogels can retain 94% of their capacitance after 5000 cycles [55], and Co_3_O_4_@HPCA-700–800 shows 92.4% capacitance retention after 10,000 cycles [60]. This stability is due to their well-defined pore structures and stable electronic interactions.

Regarding thermal and oxidation stability, carbon aerogels feature low thermal conductivity (<0.02 W·m^−1^·K^−1^) and high heat resistance, making them suitable for flame retardancy and thermal insulation applications [107,108]. Their carbon matrix also exhibits good oxidation resistance under inert atmospheres during carbonization, maintaining structural integrity at high temperatures [32].

## 4. Application of Carbon Aerogel

Carbon aerogel can be used for adsorbing grease and heavy metal ions in sewage and the atmospheric environment in the adsorption field. It also has good applications in the sensor field, and is particularly widely used in electrode materials. Its applications in the electrochemical field have been extended to many aspects, such as secondary batteries, fuel cells, supercapacitors, and electrocatalysis.

### 4.1. Electrochemistry

#### 4.1.1. Supercapacitors

In Second 2 of this paper, the basic principles of energy storage capacitors and the relationship between different application requirements and CAs performance characteristics are introduced in detail. Therefore, they will not be detailed here.

#### 4.1.2. Capacitive Deionization

Capacitive deionization (CDI), an innovative and energy-efficient electrochemical desalination technology, operates through an electrosorption mechanism where ions are adsorbed onto the electric double layers (EDLs) of porous carbon electrodes under low applied potentials (0.6–2.0 V DC). In this process, negatively charged cathodes selectively adsorb cations such as Ca^2+^, Mg^2+^, and heavy metal ions (e.g., Cr^3+^, Pb^2+^, Cd^2+^), while positively charged anodes capture anions including NO_3_^−^, SO_4_^2−^, Cl^−^, and AsO_4_^3−^, enabling effective water deionization and purification. As a non-Faradaic process, CDI relies fundamentally on ion electrosorption at charged carbon surfaces [109].

The pore architecture of carbon aerogels critically determines their capacitive deionization (CDI) performance, where micropores (0.5–2 nm) enable efficient ion adsorption through electric double-layer (EDL) formation, as demonstrated by Cheng et al. [110] who observed that KOH-activated carbon aerogels with 70% micropore content achieved 16 mmol/g CO_2_ adsorption under 30 bar at 25 °C due to enhanced surface charge density. Concurrently, mesopores (2–50 nm) facilitate rapid ion diffusion kinetics, as exemplified by Yang et al. [102], in whose study Co_3_O_4_/carbon aerogel composites containing 40% mesopores exhibited a specific capacitance of 1310 F/g, outperforming microporous counterparts. Beyond pore size distribution, ion selectivity can be enhanced through surface modifications such as N-doping, which introduces polar functional groups (e.g., pyridine-N) to preferentially adsorb target ions like Pb^2+^, Zang et al. [111] reported that chitosan/graphene composite aerogels (CS/GCA) with 5 wt% graphene oxide achieved Pb^2+^ adsorption capacities of 100 mg/g—threefold higher than unmodified carbon aerogels. Furthermore, hierarchical pore networks synergistically improve electrode durability by mitigating structural decay, as evidenced by Qian et al. [2], who reinforced carbon aerogels with embedded carbon fiber fabrics, elevating cycling stability from 83% to 99% through mechanical reinforcement.

Recent advancements have expanded CDI applications through novel electrode materials, with Moreno-Castilla and Maldonado-Hódar [112] pioneering developments in aerogel-based electrodes. Emerging studies systematically investigate ionic adsorption mechanisms in CDI systems utilizing aerogel electrodes, with Yang et al. [113] extending EDL models to porous aerogel architectures in aqueous solutions. Notably, silica gel–aerogel composite electrodes have been engineered and characterized through electrochemical analyses, demonstrating optimal performance metrics [114]. Resorcinol–formaldehyde (RF) organic aerogel electrodes exhibit exceptional properties, achieving electrical conductivity of 13.2 S/cm, specific capacitance of 220 F/g, and 97.6% salt removal efficiency at 1.7 V [115].

A critical challenge for nanostructured aerogel electrodes lies in their susceptibility to contamination from brackish water impurities during deionization. Recent studies have investigated this stability limitation [116,117], with Xu et al. [117] specifically analyzing how operational parameters influence CDI system performance in brackish water environments. These investigations highlight the importance of optimizing electrode materials and process conditions to mitigate fouling while maintaining desalination efficiency.

Carbon aerogel materials exhibit significant potential for application in the capacitive deionization (CDI) field, owing to their high specific surface area, well-developed pore structure, and excellent electrical conductivity coupled with efficient electron transport capabilities. The utilization of biomass-derived precursors further endows them with environmental friendliness and renewability. However, challenges persist in practical implementation, including complex fabrication processes with elevated costs, insufficient mechanical and cycling stability, etc. Future research efforts should focus on structural refinement, the development of green preparation processes, and multifunctional integration strategies to overcome these limitations. These advancements are expected to promote sustainable development in water treatment technologies.

#### 4.1.3. Electrocatalysis

Under the condition that the types of catalysts are the same, the performance of catalysts is mainly affected by the effective catalytic area and the catalyst loading amount [118,119,120]. For practical applications, it is crucial to ensure sufficient contact between the active sites of the catalyst and the reaction products. Therefore, selecting an appropriate catalyst carrier is particularly important. Among numerous candidate carrier materials, carbon aerogel has obvious advantages as a catalyst carrier material [121,122].

**Figure 6:**
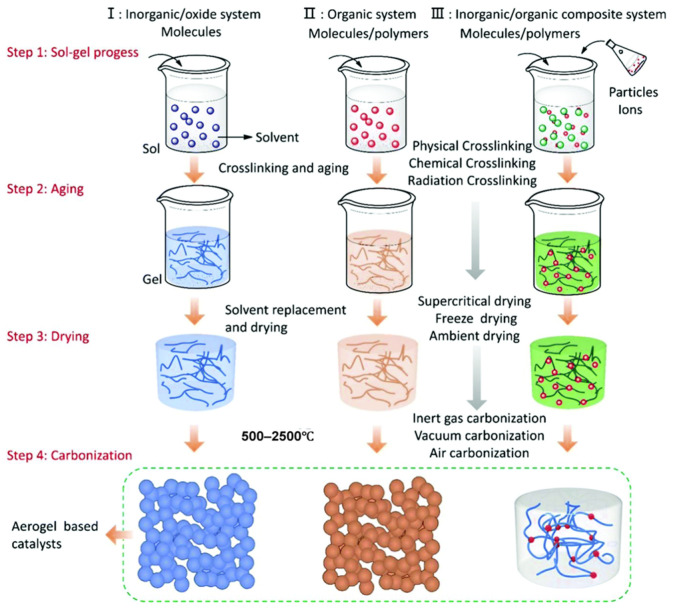
The synthetic strategies of aerogel-based catalysts. Reprinted with permission from [125].

This is because during the synthesis process of carbon aerogel, the catalyst can be in situ compounded into the carbon skeleton (Figure 6), and due to its three-dimensional porous structure, it can isolate metal atoms and suppress agglomeration before high-temperature sintering. Secondly, compared with ordinary particulate catalysts, carbon aerogel catalysts provide more active sites, enabling the catalyst to be utilized more fully. In addition, carbon aerogel can also be fabricated into integral structures, flexible, or special-shaped structures, and it can be used without the need for an additional current collector, which will greatly reduce the overall mass of the catalyst device and can also improve the flexural resistance performance of the catalytic electrode. Compared with traditional inorganic aerogels, the high electrical conductivity of carbon aerogel makes it an ideal material for preparing electrocatalyst carriers and supercapacitors [87,123,124]. Different catalytic systems have different requirements for catalyst carriers. Electro-catalysis prefers substrates with high electrical conductivity, while photocatalysis requires substrates with light gaps. In addition, there are some other characteristics, such as mechanical properties, pore structure, hydrophobicity, biocompatibility, transparency, and thermal conductivity, which can meet the needs of different fields. In applications, the catalyst carrier should be selected according to the specific usage scenarios to achieve the best catalytic effect.

After the sol–gel process treatment, the skeletal structure of the aerogel catalyst is completely formed. Compared with traditional catalyst material synthesis methods (such as the hydrothermal method, co-precipitation method, and liquid-phase reaction method), the aerogel-based catalyst synthesis method is more direct and can incorporate multiple molecular, ionic, and particle precursors in situ. The carbon aerogel is obtained by carbonizing organic aerogel or using carbon materials, such as graphene and carbon nanotubes, as precursors. A functionalized aerogel is formed by compounding specific functional substances with the porous skeleton of aerogel [126,127,128,129,130]. For aerogel materials with catalytic functions, supported catalysts can be selected as single-component, two-component, or multi-component according to the requirements. To ensure that the precursors react fully, the wet gel obtained through the sol–gel process needs to be further aged to enhance the three-dimensional skeletal structure of the gel. Combined with some reports in the literature, Figure 6 summarizes the current preparation routes of various composite aerogel catalysts [131,132,133,134,135,136].

For carbon-based aerogels, the carbonization process after drying is usually carried out under the protection of inert gas at a temperature above 500 °C. As the temperature rises, organic substances are pyrolyzed, and hydrogen and oxygen in the polymer gradually volatilize, leaving a porous carbon skeleton. Meanwhile, the supported catalyst is reduced and crystallized at high temperatures. Figure 6 shows the synthesis process of organic-inorganic composite aerogel. Before the carbonization process, one or more types of molecules, ions, or particles can be directly compounded into the aerogel, and in this case, the physical properties of the aerogel are basically not affected. For example, Gu et al. [88]. used (Ni^2+^, Fe^3+^)/sodium alginate/reduced graphene oxide (r-GO) hydrogel as a precursor and prepared a 3D Ni_3_FeN/r-GO aerogel electrocatalyst by the ion exchange method. The catalyst exhibited good hydrogen evolution reaction (HER) and oxygen evolution reaction (OER) activities, as well as stable performance. In addition, the prepared aerogel can also be loaded with catalysts by methods such as soaking the solid aerogel in a metal salt solution or vapor deposition. These methods are considered to be the simplest and most economical methods for preparing various composite catalysts. In recent years, the development of organic–inorganic composite catalysts has brought great opportunities for applications such as photocatalysis, electrocatalysis, and heterogeneous catalysis. The introduction of new concepts such as quantum dots and single atoms has also brought new potential to aerogel catalysts. The development technologies of these new types of aerogel catalysts will surely push the entire catalytic field to a new stage. Li et al. [137] presents a significant advancement in electrocatalyst design through the development of N-doped carbon dots confined PtNi alloy aerogels (NCDs-PtNi aerogels). By integrating NCDs into the hierarchical porous PtNi framework, the composite aerogel achieves exceptional structural stability and modulated electronic properties, including a downshifted d-band center and enhanced vacancy formation energies. This design yields outstanding bifunctional performance for both methanol oxidation reaction (MOR) and oxygen reduction reaction (ORR) in acidic media. Notably, the NCDs-PtNi aerogels demonstrate a 12-fold increase in specific activity over commercial Pt/C for MOR, coupled with dramatically enhanced durability—retaining 52% of initial activity after 5000 cycles, far exceeding Pt/C (15%) and unmodified PtNi aerogels (10%). Similarly, for ORR, they exhibit 5.6 times higher specific activity than Pt/C and maintain 87% activity after 50,000 seconds. The NCD confinement effectively improves CO tolerance, lowers reaction energy barriers, and mitigates active site degradation, establishing a feasible strategy for creating robust, high-performance electrocatalysts critical for practical methanol fuel cell applications.

Due to the numerous advantages of aerogel catalysts, such as large specific surface area, adjustable porosity, high efficiency in separating metal atoms, and good chemical/physical stability, their applications in many fields, such as photocatalysis, electrocatalysis, homogeneous catalytic reactions, fuel cells, Li-O_2_ batteries, and zinc-ion batteries, have attracted attention. Scientists have conducted systematic research on their reaction mechanisms by developing new material systems and relying on new equipment to further improve the electrochemical kinetics and stability of catalysts. In the early stage of research, noble metal-based catalysts were widely studied due to their excellent performance, but their high cost is a challenge for large-scale commercialization. In recent years, research has focused on transition metal catalysts. By compounding them with rare elements, their cost can be balanced and their stability can be promoted.

The catalytic performance of catalyst materials is affected by the intrinsic properties of catalyst elements and surface effects. The intrinsic properties of catalyst elements determine the electronic structure effect of catalyst elements, and this effect is mainly limited by the energy band and surface state density of the elements. The surface effect of the catalyst mainly refers to the influence of the electronic interaction on the surface and interface layers between the catalyst material (surface chemical structure and atomic arrangement structure) and the reaction system on the catalytic rate. Both factors have a great impact on the performance of the catalyst. Among them, the influence of the choice of elements on the catalytic activity can reach dozens of orders of magnitude, and the influence of the surface structure effect of the catalyst is within 1–2 orders of magnitude. In the actual operation of the catalyst, they are not completely independent but have a synergistic effect. Even so, when selecting a catalyst material system, priority should be given to the electronic structure effect. By selecting an appropriate material system, catalysts with low power consumption and high activity can be obtained.

In addition to the choice of elements, the catalyst loading amount is also a key factor affecting performance. When considering cost and sustainable development, a relatively low metal content should be maintained. However, an overly low metal content may lead to insufficient catalytic activity. In many catalytic reactions, although the deficiency of low metal loading can be compensated by increasing the amount of catalyst used. However, this method of simply increasing the amount of catalyst can only improve the catalytic activity to a certain extent. Moreover, as the amount of catalyst increases, the specific activity of the catalyst gradually decreases. For example, in proton exchange membrane fuel cells (PEMFC), although excessive use of the catalyst can compensate for the performance loss caused by low metal loading, a thick catalyst layer will lead to mass transfer loss and then result in a loss of power density.

**Figure 7:**
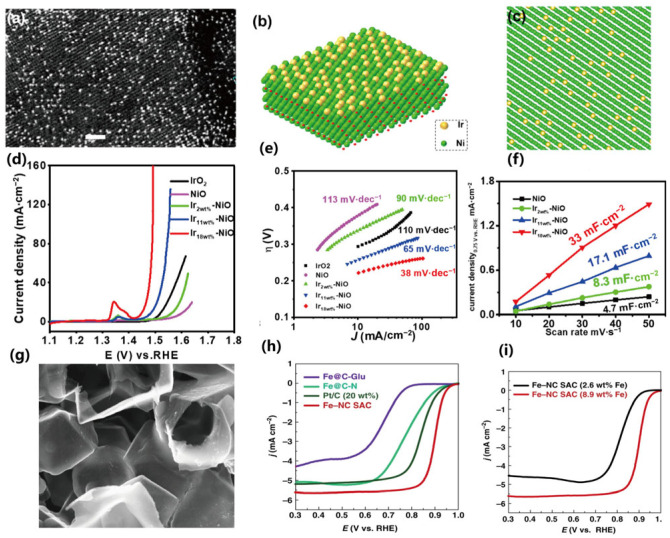
High-loading catalysts with different metal contents and their performance comparison. (**a**) HAADF-STEM micrograph of the Ir-NiO catalyst; (**b**,**c**) corresponding atomic models; (**d**,**e**) polarization curves and Tafel plots of OER; (**f**) estimated value of double-layer capacitance. Reprinted with permission from [118]. (**g**) SEM image of the porous carbon framework; (**h**) steady-state ORR polarization curves of different SACs; (i) steady-state ORR polarization curves of the catalyst under different Fe loading amounts. Reprinted with permission from [119].

The unique advantage of high-loading catalysts is that they can maintain a high specific activity of the catalyst. Wang et al. [118]. recently reported a two-step wet chemical method for synthesizing Ir-NiO/carbon cloth catalyst, with the metal loading increased to 18 wt% (Ir on NiO), as shown in Figure 7.

The oxygen evolution reaction performance of the obtained single-atom catalysts (SACs) is significantly improved with the increase in metal content. Zhao et al. [119]. reported the synthesis of single-atom catalysts by using the Cascade Anchoring Strategy and achieved a high metal loading of up to 12.1%. The high-loading catalysts exhibited better electrocatalytic performances in oxygen reduction and carbon dioxide reduction reactions. Based on this, we also proposed a synthesis method for high-loading catalysts by combining the host–guest inclusion effect in the self-healing effect, utilizing the host–guest interaction between cyclodextrin and ferrocene to achieve the atomic-level spatial confinement effect, so as to realize the atomic independence throughout the entire catalyst synthesis process. This method can theoretically achieve a single-atom loading of over 6 wt% [125].

Due to the high specific activity of high-loading catalysts, the amount of catalyst used can be significantly reduced, and the mass of the entire device can be decreased, thereby improving the energy density and power density of the device. In addition, high-loading catalysts are also important for establishing the synergistic effect among elements. By developing ligands, metal atoms with different valence states can act synergistically. By reducing the probability of contact between target atoms, influencing the crystallization process of a single element or reducing the crystal size, etc., the leaching or the formation of nanoparticles can be prevented. In this way, the gap between homogeneous and heterogeneous catalysis can be bridged. The controllable synthesis of high-loading single-atom catalysts will be an important direction for development in the next stage.

**Figure 8:**
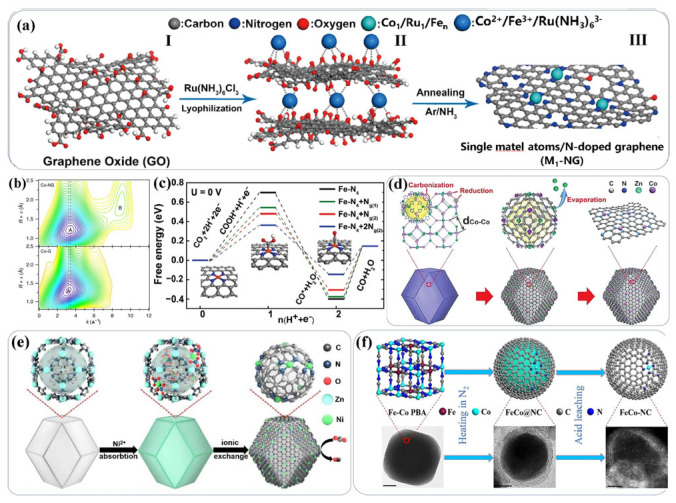
(**a**) Schematic diagram of the synthesis process of the Ru1/Co1/Fen catalyst [138]; (**b**) wavelet transform of Co1-NG and Co-G. Reprinted with permission from [139]. (**c**) Theoretical calculation and proposed mechanism of the nitrogen-coordinated iron catalytic center. Reprinted with permission from [140]. (**d**–**f**) Schematic diagrams of the synthesis processes of some single-atom catalysts. Reprinted with permission from [141,142,143].

In addition to traditional experimental research methods, with the deepening of research, some new characterization methods and synthesis methods have also been gradually developed (Figure 8).

New characterization methods that have emerged in recent years include synchrotron radiation and spherical aberration corrected electron microscopy. New methods such as density functional theory (DFT) calculations and materials genomics can also be used for high-throughput screening in the in-depth simulation of material systems. Meanwhile, they can be combined with new technologies brought about by deep learning and machine learning in the field of artificial intelligence. Thanks to the emergence of new technologies like synchrotron radiation and spherical aberration–corrected electron microscopy, single-atom catalysts can be successfully characterized. For example, Tour et al. [138,139] annealed reduced graphene oxide (rGO) and a small amount of Co, Ru, and Fe salts in a gaseous NH_3_ atmosphere to obtain nitrogen-doped graphene (NG) catalysts. Through extended X-ray absorption fine structure (EXAFS) analysis at the K-edge, the existence of single-atom Co was proved. Electrochemical tests showed that this material exhibited good hydrogen evolution reaction (HER) activity. By expanding this method, a series of single-atom materials such as Fe, Co, and Ni were synthesized. In addition, some new synthesis methods, such as selecting appropriate organic ligands as templates, can also achieve good single-atom synthesis results, as shown in Figure 8d–f [141,142,143]. With the deepening of research and the development of technology, the difficulties faced by high-performance catalysts will be solved one by one, and the potential of high-loading catalysts will be further highlighted.

### 4.2. Water Treatment

#### 4.2.1. Oil/Water Separation

Currently, three primary methodologies exist for oil/water separation: physical, chemical, and biological technologies. The chemical approach typically employs reactive agents to treat oil/water mixtures or facilitates direct combustion. However, this method presents significant drawbacks including secondary pollution risks such as CO_2_ emissions from combustion processes and potential contamination from residual chemical agents. While biological methods utilize organic materials for pollutant degradation without secondary contamination, their practical application is constrained by high operational costs and limited economic viability. In contrast, physical adsorption has emerged as the most effective separation technique due to its environmental sustainability, material recyclability, and operational simplicity through the use of porous materials [144,145,146,147,148]. This method enables rapid and efficient phase separation through straightforward adsorption processes. Nevertheless, conventional adsorbents like activated carbon and zeolites exhibit limited absorption capacities and complex post-adsorption recovery requirements. Aerogels have consequently gained prominence as superior absorbent materials for large-scale oil/water separation, offering enhanced cost-effectiveness through simplified oil recovery via mechanical squeezing.

Recent advancements demonstrate the development of high-performance composite aerogels. He et al. [149] fabricated bacterial cellulose/silica composite aerogels through the surface deposition of silicon particles followed by freeze-drying with aging and solvent exchange processes. The resultant material exhibited exceptional characteristics including ultralow density (0.031 g/cm^3^), high specific surface area (734.1 m^2^/g), minimal thermal conductivity (0.031 W/m·K), and superior oil absorption capabilities coupled with facile water separation. An alternative silicon-based aerogel prepared via vacuum filtration of cellulose aerogel impregnated in silicon solution demonstrated remarkable super-elasticity with 88% oil recovery efficiency.

Innovative biomass-derived aerogels have also shown promising results. Li et al. [104] synthesized carbon aerogels from winter melon through hydrothermal processing at 180 °C for 10 h in a Teflon-lined autoclave, followed by impurity removal and cleavage. The resulting aerogel achieved ultralow density (0.048 g/cm^3^), pronounced hydrophobicity, and exceptional absorption capacities of 16–50 times its weight for various oils and organic solvents. Similarly, Wang et al. [105] developed carbonaceous aerogels from lettuce via a 10 h hydrothermal treatment at 180 °C, subsequent purification, and vacuum freeze-drying. Post-modification with polydimethylsiloxane (PDMS) coating enabled integration with specialized oil collection systems, facilitating continuous oil recovery from both water surfaces and submerged environments through pump-assisted operations, surpassing conventional sorbent materials in operational efficiency.Sun et al. [150] presents an innovative, environmentally friendly superhydrophobic adsorbent (PPCA@PFTS) fabricated by modifying biomass-derived pomelo peel carbonaceous aerogel (PPCA) with perfluorooctyl-trimethoxysilane (PFTS). The resulting material features a unique 3D cross-staggered architecture with macro-tunnels and mesopores, endowing it with exceptional oil/organic solvent sorption capacity (up to 5.8 g/g for soybean oil), ultra-high hydrophobicity, and rapid separation kinetics. Critically, PPCA@PFTS enables practical water remediation through its compatibility with self-priming pump systems for swift contaminant collection and exhibits outstanding recyclability—maintaining high performance through 30 regeneration cycles without significant degradation in sorption capacity or contact angle. This combination of sustainable biomass sourcing, straightforward fabrication, operational efficiency, and cyclic stability positions PPCA@PFTS as a technologically advanced and economically viable solution for oil spill cleanup and organic pollutant removal from water surfaces.

#### 4.2.2. Removal of Heavy Metal Ions

**Figure 9:**
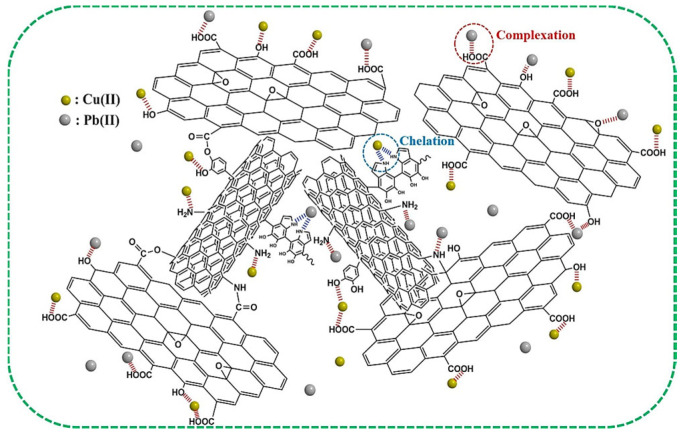
The complexation (red) and chelation (blue) interaction of Pb(II) and Cu(II) adsorption on MWCNT-PDA/GO hybrid aerogel. Reprinted with permission from [111].

Adsorption has emerged as a prominent sewage treatment technology due to its cost-effectiveness, operational simplicity, substantial adsorption capacity, and high removal efficiency [151]. The adsorption selectivity and efficiency of aerogels are fundamentally governed by their surface characteristics and chemical properties, including structural morphology, hydrophobicity, and polarity [152,153]. Recent investigations by Zang et al. revealed that porous CS/GCA structures exhibit enhanced lead ion (Pb^2+^) adsorption capabilities, with adsorption capacity increasing from 68.5 to 100 mg·g^−1^ when graphene oxide (GO) content reached 5 wt% in the aerogel matrix. The adsorption mechanism for common heavy metal ions such as Pb^2+^ and Cu^2+^ primarily involves intergroup coordination and complexation reactions, as illustrated in Figure 9 [111].

Adsorption technology demonstrates significant advantages for treating both oily wastewater and heavy metal-laden effluents, owing to its operational efficiency, simplicity, and environmental compatibility. For oil/water separation, physical adsorption using advanced aerogels—particularly ultralight, hydrophobic biomass-derived composites with high adsorption capacities—overcomes the limitations of conventional chemical and biological methods by enabling efficient phase separation and facile recovery. This is exemplified by winter melon-derived carbon aerogel (density: 0.048 g/cm^3^), which achieves exceptional organic solvent adsorption exceeding 1000 mg/g [104]. In heavy metal ion removal, adsorption efficacy depends critically on adsorbent surface properties (e.g., pore structure, functional groups), leveraging coordination/complexation mechanisms for selective ion capture. Representative materials like N-doped CS/GCA aerogel exhibit high performance for Pb^2+^ removal (>200 mg/g adsorption capacity) [111].

### 4.3. Other Applications

#### 4.3.1. Flame Retardancy and Thermal Insulation

Thermal insulation plays a pivotal role in enhancing building energy efficiency by minimizing thermal energy losses in heating/cooling systems. High-performance insulation materials demand low thermal conductivity, sustainability, cost-effectiveness, and environmental compatibility. Carbon aerogels, with their hierarchical 3D porous structure, ultralow density (<0.1 g/cm^3^), and exceptional thermal conductivity (<0.02 W·m^−1^·K^−1^), surpass conventional materials like expanded polystyrene and mineral wool. Their bio-based nature and scalable production further position them as sustainable alternatives.

Liu et al. [154] developed lightweight ceramizable phenolic aerogel/carbon fiber (PA/CF) composites modified with TiB_2_ and B_4_C via an acid-catalyzed sol–gel process, achieving rapid gelation for uniform filler distribution. The composites exhibit exceptional flame-retardant and thermal protection properties derived from synergistic high-temperature reactions of TiB_2_/B_4_C, which consume oxygen, block oxygen diffusion, and fix carbon, coupled with the formation of a protective multiphase ceramic barrier (C-B_2_O_3_-TiO_2_-TiC). This unique ceramization mechanism endows the material with outstanding thermal stability (58.8% residual weight at 1200 °C), oxidation resistance (3.44 MPa residual strength post-static ablation), and ablation resistance (linear ablation rate: 0.045 mm/s; mass ablation rate: 0.0115 g/s), representing significant improvements over unmodified composites (e.g., 57.73% higher residual carbon, 6-fold higher post-ablation strength, 44.4% lower linear ablation). Combined with inherent lightweight characteristics (0.452–0.608 g/cm^3^), high strength (13.86 MPa), and low thermal conductivity (0.133–0.250 W·m^−1^·K^−1^), these composites demonstrate high competitiveness for thermal protection systems in hypersonic vehicles.Zuo et al. [155] pioneers the rapid visible-light vat photopolymerization (VP) 3D printing of fully biomass-based hydrogels, derived from renewable itaconic acid, gelatin, and sodium alginate, using a commercial LCD printer. The resulting covalently cross-linked networks exhibit a highly ordered layer-by-layer structure, which critically endows them with exceptional flame-retardant properties. These include an outstanding limiting oxygen index (LOI) of 83.5%, surpassing previously reported biomass hydrogels, alongside remarkable temperature resistance and advantageous combustion behaviors. This combination of superior flame retardancy and mechanical strength, achieved through sustainable materials and energy-efficient manufacturing, establishes a green and precise platform for developing next-generation flame-retardant materials with significant industrial application potential.

Current research trends, as summarized in Table 7, reveal concentrated efforts on nanocellulose-based aerogels and lignocellulosic composites for thermal management applications. Studies consistently demonstrate that cellulose aerogels derived from wood, cotton, and agricultural residues achieve thermal conductivity values competitive with petroleum-based insulators, while offering advantages in renewability and carbon sequestration potential. These developments highlight the transition toward bio-based, multifunctional insulation materials in sustainable architecture.

#### 4.3.2. Microwave Absorption

The proliferation of GHz-band electromagnetic pollution necessitates high-performance microwave absorption materials (MAMs). Carbon-based absorbers, recognized for their low density, tunable conductivity, and environmental stability, face challenges in achieving broadband absorption due to impedance mismatch and reliance on single attenuation mechanisms [166]. Two primary strategies address these limitations: (1) blending carbon with magnetic materials to enhance permeability [167,168,169,170], though this introduces high density, corrosion susceptibility, and filler dependency (>30 wt%) [171,172]; and (2) engineering porous architectures to reduce permittivity and optimize impedance matching while enabling multi-scale attenuation via interfacial polarization and microwave scattering [173,174,175].

The pursuit of high-performance electromagnetic (EM) wave absorbers demands innovative strategies to overcome fundamental limitations such as narrow bandwidth, impedance mismatch, thickness constraints, and directional sensitivity. Recent breakthroughs leverage advanced structural engineering of lightweight aerogels, transforming these porous materials into highly efficient EM dissipative platforms. By precisely controlling material architecture across multiple scales—from nano/microscale alignments to macroscopic assemblies—researchers are developing a new generation of aerogel-based absorbers. These designs synergistically enhance impedance matching, optimize energy dissipation pathways, and mitigate anisotropy, achieving unprecedented combinations of ultrawide bandwidth, minimal thickness, low density, and angular stability. These pioneering approaches exemplify this paradigm shift, where tailored structural ingenuity unlocks exceptional EM absorption performance previously unattainable with conventional materials.

Wang et al. [176] pioneered ultra-broadband electromagnetic (EM) absorption aerogels through multiscale structural engineering, utilizing electric and temperature fields to orchestrate hierarchical architectures. At the microscale, bidirectional MXene/polyimide aerogels (BAs) feature vertically aligned Ti_3_C_2_Tₓ nanosheets and layered walls perpendicular to incident waves, facilitating efficient electromagnetic-to-electrical energy conversion and achieving an 8.58 GHz effective absorption bandwidth (EAB) at 2.1 mm thickness. At the macroscale, non-gradient multilayer stacking of BAs resolves the fundamental conflict between impedance matching and energy dissipation while amplifying interfacial reflections, extending EAB to 9.41 GHz at 3 mm thickness—among the broadest reported. This synergistic structural control across scales (micro-alignment for conduction loss vs. macro-assembly for optimized impedance) enables record-breaking performance at ultralow density (≈20 mg cm^−3^), establishing a groundbreaking paradigm for designing ultrathin, high-efficiency EM absorbers. Subsequently, in subsequent research, Wang et al. [177] overcomes the limitations of conventional aerogels in electromagnetic wave (EMW) absorption by developing MXene-based aerogels with hierarchically engineered structures through innovative freezing techniques. Dual-scale porous structures—featuring synergistic micrometer-scale (≈3 µm) pores within walls and submillimeter-scale (≈200 µm) pores between walls—were fabricated via constant-temperature freezing of MXene/TPU solutions, achieving record broadband absorption with an ultra-wide effective absorption bandwidth (EAB) of 10.41 GHz and minimum reflection loss (RL_min_) of −52.41 dB under vertical incidence due to enhanced energy dissipation. To address severe directional sensitivity, a secondary-infusion freezing method was employed to construct a dual-network architecture, introducing perpendicular reflective interfaces that dramatically improved parallel-direction absorption—increasing EAB by 275% (from 1.58 to 5.93 GHz) while maintaining high vertical direction performance (8.08 GHz EAB). This multiscale structural design paradigm resolves intrinsic conflicts between anisotropy and omnidirectional absorption, significantly reducing EMW incidence-angle dependence and establishing a versatile strategy for high-performance absorbers in complex electromagnetic environments.

Liu et al. [178] demonstrates the strategic integration of 3D printing via direct ink writing (DIW) to fabricate ultralight MXene/reduced graphene oxide (rGO) aerogel frames (MGs) that overcome the intrinsic impedance mismatch limitation of highly conductive MXene for electromagnetic wave absorption (EWA). By engineering low-concentration emulsion inks and optimizing compositional ratios alongside programmable structural design, the printed aerogels achieve exceptional EWA performance: an effective absorption bandwidth (EAB) of 8.10 GHz (spanning most of the X-band and the entire Ku-band) and a minimum reflection loss (RL_min_) of −56.85 dB. Furthermore, the DIW-architected aerogels simultaneously deliver robust mechanical integrity and thermal insulation capabilities. This work establishes DIW as a versatile manufacturing paradigm for structurally tailored electromagnetic absorption (EMA) composites, providing critical insights for future high-performance multifunctional material design.

#### 4.3.3. Carbon Capture

Carbon capture and storage (CCS) technologies are pivotal for mitigating greenhouse effects, with CO_2_ capture serving as a prerequisite for subsequent conversion or sequestration [179]. Among capture methods, adsorption is considered economically viable due to its low energy consumption, recyclability, and efficiency in low-concentration CO_2_ scenarios [180,181,182]. Carbon aerogels (CAs), characterized by their three-dimensional porous structures, high porosity (80–90%), tunable pore sizes (2–50 nm for mesopores), and large specific surface areas, have emerged as promising adsorbents for CO_2_ capture [81,183,184,185,186,187]. Key factors influencing CA performance include pore structure (micropores dominate low-pressure physisorption, while mesopores enhance kinetics), surface chemistry (heteroatom doping improves selectivity), and activation methods (e.g., KOH increases surface area but risks pore collapse) [103,106,110].

Cheng et al. [110] developed a KOH-activated carbon aerogel (CA-KOH) from Typha Orientalis biomass via freeze-drying and carbonization. The activation process enlarged pores, increased the specific surface area to 1841.89 m^2^/g, and achieved a CO_2_ adsorption capacity of 16 mmol/g at 25 °C and 30 bar. The material also demonstrated multifunctionality, enabling sequential hydrogen storage (0.61 wt% at 80 bar), CO_2_ capture, and VOC removal (e.g., 123.31 mg/g for o-xylene) without requiring intermediate desorption [110]. Geng et al. [106] synthesized lignin/cellulose nanofiber-derived carbon aerogels (LTCAs), where adjusting the lignin-to-CNF ratio optimized pore structure and surface area, yielding a CO_2_ adsorption capacity of 3.39 mmol/g at 0 °C and 1.0 bar. However, sodium salt residues were found to block mesopores and micropores, underscoring the importance of precursor purification [106]. Li et al. [103] further enhanced CO_2_ adsorption (3.65 mmol/g at 25 °C and 1.0 bar) by nitrogen doping via urea modification during cellulose aerogel synthesis, which increased surface polarity and CO_2_ selectivity [103].

## 5. Conclusions and Perspective

Carbon aerogels, as a novel class of nanoporous materials, have demonstrated tremendous potential in energy and environmental applications owing to their three-dimensional interconnected network architecture, tunable pore topology, ultra-high specific surface area, and exceptional electrical conductivity–flexibility synergy. The current research has revealed that their hierarchical pore systems significantly optimize mass transport kinetics through synergistic effects of micropore–mesopore–macropore interactions, while surface chemical modification strategies (including heteroatom doping and metal nanoparticle hybridization) effectively regulate electronic structures, endowing the materials with dual energy storage characteristics and catalytic activity. Particularly in supercapacitor applications, breakthrough achievements in energy–power density balance have been realized via the cooperative enhancement of electric double-layer capacitance and pseudo-capacitance. Although biomass-derived carbon aerogels have driven innovations in sustainable fabrication technologies, existing synthesis systems remain constrained by challenges including strong feedstock specificity, high energy consumption in processing, and difficulties in cost control during scale-up.

Future advancements in this field are anticipated to witness multidimensional breakthroughs. At the fundamental mechanism level, density functional theory (DFT) calculations will be strategically employed to elucidate the dynamic electronic structure evolution of heteroatom-doped active sites, establishing structure–property correlation models to guide the spatial orientation of high-density active sites. In terms of fabrication process innovation, artificial intelligence technologies will be deeply integrated into material development workflows. Machine learning models will decode complex nonlinear relationships between synthesis parameters (e.g., precursor characteristics, pyrolysis temperature, and activating agent ratios) and material performance metrics, enabling reverse design of pore architectures, doping configurations, and mechanical properties. From a sustainability perspective, advanced resource utilization technologies for organic solid wastes (agricultural residues, textile waste, etc.) require focused development. Component separation and catalytic pyrolysis pretreatment approaches will be optimized to regulate the decomposition pathways of biomass constituents (cellulose/hemicellulose/lignin), constructing inherently doped hierarchical porous carbon frameworks. Regarding application expansion, traditional functional limitations will be transcended through 3D printing technologies to fabricate flexible freestanding electrode–catalyst integrated devices. These innovations will enable intelligent systems combining capacitive deionization, electrocatalytic degradation, and energy storage functionalities, accelerating the transition from laboratory research to industrial implementation. Carbon aerogels are poised to evolve into advanced platform materials integrating the structure–function–application trinity. Such materials are expected to play pivotal roles in carbon neutrality strategies by providing disruptive solutions for constructing high-efficiency energy storage systems and achieving resource-oriented pollutant treatment. 

## Figures and Tables

**Table 1:** Summary of the porous carbon material synthesis strategies [33,34].

Methods	Processes	Conditions	Temperature (°C) ^a^	Structure
Carbonization	Direct pyrolysis	N_2_ flow and high temperature	>500	Inherent morphologies with small surface area
	Hydrothermal carbonization	Generally combined with carbonization or activation	120–250	High graphitization
Activation	Physical activation	CO_2_, steam, O_2_, etc.	600–1200	Large surface area up to 3000 m^2^ g^−1^
	Chemical activation	KOH, KHCO_3_, ZnCl_2_, H_3_PO_4_, NaOH, FeCl_3_, etc.	400–1000	Large surface area up to 3000 m^2^ g^−1^
Template methods	Hard template	Silica; metal oxides (MgO, ZnO, etc.); molten salts (NaCl, KCl, LiCl, etc.)		Well-designed structure with ordered morphology and adjustable porosity

Note: ^a^ The temperature in the table is a range value, reflecting the fluctuation range of process conditions. The structural characteristics are qualitative descriptions without quantitative uncertainty.

**Table 2:** Different activation methods for preparing porous carbon materials and the characteristics.

Processes	Activating Agent	Activation Method	BET Surface Area (m^2^ g^−1^) ^a^	Pore Volume (cm^3^g^−1^) ^b^	Ref.
Physical activation	Steam	Pine nut shell; carbonization at 500 °C for 15 mins, activation at 900 °C for 75 min	956 ± 20	0.620 ± 0.012	[38]
	CO_2_	Banana flesh; carbonization at 300 °C for 1 h, activation at 900 °C for 4 h	1415 ^c^ ± 30	0.746 ± 0.015	[39]
Chemical activation	KOH	Soybean; carbonization at 800 °C for 2 h, KOH/carbon material = 3:1, activation at 700 °C for 2 h	1749 ± 30	-	[40]
	NaOH	Waste mangosteen peel; carbonization at 600 °C for 2 h, NaOH/carbon material = 7:2, activation at 700 °C for 2 h	2623 ± 50	-	[41]
	K_2_CO_3_	K_2_CO_3_/carbon material = 5:2, activation at 850 °C for 1 h	2312 ± 50	2.807 ± 0.056	[42]
	ZnCl_2_	Aerobio granular sludge; 5 mol/L ZnCl_2_ solution for 24 h, carbonization at 700 °C for 2 h	852 ^d^ ± 20	0.086 ± 0.002	[43]
	H_3_PO_4_	Baobab fruit shells; hydrothermal carbonization at 160 °C for 16 h, activation at 800 °C for 2 h	912 ^e^ ± 20	0.470 ± 0.009	[44]

Note: ^a,b^ The uncertainty of the BET surface area and pore volume in the table is estimated based on repeated data from experiments in the literature, and is calculated at a value of ±2% if not explicitly labeled. ^c^ The value reported in the literature is 1414.97. This value was rounded to the nearest integer. ^d^ The value reported in the literature is 852.41. This value was rounded to the nearest integer. ^e^ The value reported in the literature is 911.7. This value was rounded to the nearest integer.

**Table 3:** Characterization techniques for carbon aerogels.

Technique	Purpose	Pros	Cons
BET	Surface area/pore size distribution	Quantifies SSA, pore volume, pore size distribution	Limited to pores < 300 nm; assumes idealized pore shapes
XRD	Crystallinity/phase identification	Identifies crystal phases, graphitization degree	Low sensitivity to amorphous phases; limited resolution for nanophases
SEM/TEM	Morphology/microstructure	Direct imaging of 3D network, pore hierarchy	Sample preparation artifacts; 2D projection limits 3D analysis
XPS	Surface chemistry/elemental states	Quantifies heteroatom doping (N, S, P), bonding types	Ultra-high vacuum required; surface contamination risks
Raman	Structural defects/graphitization	Evaluates disorder (D-band) vs. graphitic order (G-band)	Semi-quantitative; laser-induced heating may alter samples
EDS	Elemental mapping	Spatial distribution of dopants/metals	Limited quantification accuracy for light elements
FT-IR	Functional group analysis	Identifies oxygen groups, organic residues	Peak overlap complicates interpretation; surface sensitivity

**Table 4:** Performances of N-doped carbon aerogels as electrode material for supercapacitors.

Material	Carbon Source	Electrolyte	Specific Capacitance (F g^−1^) ^a^	Ratc Capacitance (F g^−1^) ^b^	Capacitance Retention ^c^	Ref.
N-doped CA-800	Cellulose	1 M H_2_SO_4_	225.0 at 0.5 A g^−1^	185.0 at 10.0 A g^−1^	5000 cycles (94.0%)	[55]
N-CA-600	Watermelon	6 M KOH	281.0 at 5.0 mV s^−1^	-	-	[56]
a-CA	Lignin	1 M H_2_SO_4_	189.0 at 1.0 A g^−1^	104.0 at 20.0 A g^−1^	10,000 cycles (97.4%)	[57]
NCF/NiO-2	Chitosan	2 M KOH	1074.0 at 1.0 A g^−1^	820.0 at 20.0 A g^−1^	5000 cycles (99.4%)	[58]
Cell@PPy	Cellulose	1 M H_2_SO_4_	387.6 at 0.5 A g^−1^	320.2 at 10.0 A g^−1^	10,000 cycles (92.6%)	[59]
HPCA	Seaweed	6 M KOH	260.6 at 1.0 A g^−1^	190.0 at 50.0 A g^−1^	10,000 cycles (91.7%)	[60]
Co_3_O_4_@HPCA-700-800	Seaweed	6 M KOH	1167.6 at 1.0 A g^−1^	500.0 at 50.0 A g^−1^	10,000 cycles (92.4%)	[60]
GCA	Agaric	6 M KOH	339.0 at 3.0 A g^−1^	308.0 at 10.0 A g^−1^	10,000 cycles (91.0%)	[61]
WCA	Watermelon	6 M KOH	333.1 at 1.0 A g^−1^	-	1000 cycles (96.0%)	[62]
C-10	Cellulose and liginin	6 M KOH	166.0 at 0.1 A g^−1^	83.0 at 20.0 A g^−1^	1000 cycles (98.6%)	[63]
CC27	Tannin	4 M H_2_SO_4_	387.6 at 2.0 mV s^−1^	-	-	[64]
Cell-CO_2_	Cellulose	1 M H_2_SO_4_	328.0 at 0.5 A g^−1^	213.0 at 10.0 A g^−1^	5000 cycles (96.0%)	[65]
BCA-2	Banana	6 M KOH	178.9 at 1.0 A g^−1^	-	10,000 cycles (98.0%)	[39]

Note: ^a–c^ Uncertainties for the table’s specific capacitance and rate capacitance values, estimated from literature replicate data in the literature (cyclic voltammetry and galvanostatic charge–discharge repeats), default to ±2% where unspecified. Capacitance retention exhibited a standard deviation below 2%. Data are presented to one decimal place.

**Table 5:** Samples of metal compound–modified porous carbon materials as electrode materials for supercapacitors under optimum conditions.

Carbon Source	Metal Compounds	Electrolyte	Specific Capacitance (F g^−1^) ^a^	Synthesis Method	Ref.
Willow catkins	MnO_2_	1 M Na_2_SO_4_	262.0 at 0.2 A g^−1^	Activation and hydrothermal treatment	[95]
Enteromorpha prolifera	MnO_2_	1 M Na_2_SO_4_	345.1 at 0.5 A g^−1^	Activation and post-annealing treatment	[93]
Kenaf stem	Fe_3_O_4_	0.1 M KCl	372.5 at 0.5 A g^−1^	Pyrolysis of MlL-88A on activated carbon	[92]
Cladophora glomerata	Fe_3_O_4_	3 M KCl	418.0 at 1.0 A g^−1^	Activation and hydrothermal treatment	[96]
Wheat straw	Fe_2_O_3_	3 M KOH	987.9 at 1.0 A g^−1^	Activation and post-annealing treatment	[97]
Cotton	Co_3_O_4_	6 M KOH	892.0 at 0.5 A g^−1^	Freeze-drying and calcination	[91]
Terminalia chebula fruit	Co_3_O_4_	2 M KOH	642.0 at 1.0 A g^−1^	Thermolysis	[98]
Pine cone flowers	Ni(OH)_2_	1 M KOH	916.4 at 1.0 A g^−1^	Alkali treatment, pyrolysis, and solvothermal treatment	[99]
Willow catkins	Ni(OH)_2_	6 M KOH	1568.0 at 1.0 A g^−1^	Acid treatment and hydrothermal treatment	[100]
Pomelo peel	Ni, Co and Al	2 M KOH	902.0 at 10.0 A g^−1^	Hydrothermal treatment and solvothermal treatment	[101]
Watermelon	MnO_2_	6 M KOH	49.3 at 0.5 A g^−1^	Hydrothermal treatment and thermolysis	[90]

Note: ^a^ Uncertainties for the table’s specific capacitance values, estimated from replicate data in the literature (cyclic voltammetry and galvanostatic charge–discharge repeats), default to ±2% where unspecified. Data are presented to one decimal place.

**Table 6:** Comparison of different preparation methods.

Preparation Type	Preparation Process	Performance Examples	Pros	Cons
Traditional Method	Sol–gel, drying, carbonization	Specific capacitance up to 62 F/g (aqueous electrolyte), but low porosity (~60%) [2]	-	High cost, petroleum-derived precursors
Green Synthesis	Hydrothermal carbonization of biomass	Oil adsorption up to 50× weight, but specific capacitance ~178 F/g (banana-derived CA) [39]	Sustainable, low-cost	Variable pore structure
Advanced Methods	Hard template (e.g., SiO_2_), heteroatom doping (e.g., N, P), composite	Specific capacitance up to 2883 F/g (N-CoFe_2_O_4_/CA) [14], CO_2_ adsorption 3.65 mmol/g (N-doped CA) [103]	Tailored pore structure	Complex synthesis

**Table 7:** Thermal conductivity of cellulose-based aerogels.

Different Cellulose Aerogels	Density (g cm^−3^) ^b^	Pore Size (nm)	Thermal Conductivity (W m^−1^ K^−1^) ^a^	Ref.
Natural cellulose aerogel	-	5–13	0.023–0.028	[107]
Natural cellulose aerogel with SiO_2_	0.007–0.201	-	0.029–0.037	[156]
Cellulose derived aerogel	0.012–0.033	10–100	0.018–0.028	[108]
Cellulose aerogel with SiO_2_	0.007–0.229	-	0.030–0.037	[157]
Regenerated cellulose aerogel	0.090–0.137	10–100	0.040–0.075	[158]
Regenerated cellulose aerogel with SiO_2_	0.125–0.225	-	0.026–0.033	[159]
Cellulose derived aerogel	0.050–0.109	5000–40,000	0.040–0.053	[160]
Wood based aerogel	0.032	-	0.033	[161]
Pineapple leaf/cotton-based aerogel	0.019–0.046	20,000–60,000	0.039–0.043	[162]
Cotton/natural fiber-based aerogel	0.028–0.105	-	0.036–0.0473	[163]
Spent coffee grounds/cotton/PVA	0.045	-	0.037–0.045	[164]
Cellulose/lignin-based aerogel	0.024–0.403	8–17	0.128–0.155	[165]

Note: ^a^ Thermal conductivity testing method: The thermal conductivity of aerogels was measured using a hot-filament technique apparatus. The tests were performed at atmospheric pressure in a temperature and humidity-controlled room (21–22 °C and 50% relative humidity). At least five samples per formulation were tested. ^a,b^ All values in the table were rounded to three decimal places.

## Data Availability

No data was used for the research described in the article.
